# Quality of Life Over Quantity: Conscious Refusal of Treatment and Stable Course of Gastric Signet-Ring Cell Carcinoma in a 94-Year-Old Woman

**DOI:** 10.7759/cureus.98597

**Published:** 2025-12-06

**Authors:** Aleksandr Martynenko

**Affiliations:** 1 Department of Internal Medicine, LLC “Multifunctional Medical Center” M-Clinic, Tashkent, UZB

**Keywords:** geriatric-oncology, oldest old, quality of life, shared decision-making, signet-ring cell carcinoma, stomach neoplasms

## Abstract

We report the case of a 94-year-old woman with histologically confirmed signet-ring cell carcinoma (SRCC) of the stomach (ICD-O-3.2 code 8490/3). The patient consciously declined oncological treatment and received only supportive and symptomatic care. Over a follow-up period of at least 12 months, she has remained alive, active, cognitively intact, and socially engaged. Despite the typically aggressive histological subtype, the disease followed an indolent clinical course. This case highlights the biological heterogeneity of SRCC and the importance of personalised oncogeriatric decision-making that prioritises autonomy and quality of life in the oldest-old population, consistent with contemporary European Society for Medical Oncology (ESMO)/International Society of Geriatric Oncology (SIOG) recommendations and palliative-oriented medical principles.

## Introduction

The current World Health Organization (WHO) and International Agency for Research on Cancer (IARC) classification defines signet-ring cell carcinoma (SRCC) of the stomach as a poorly cohesive adenocarcinoma. This is traditionally regarded as the diffuse type of gastric cancer described by Lauren [1,2]. SRCC is characterised by infiltrative growth, a tendency towards peritoneal dissemination and a poor prognosis in advanced stages [3]. However, population-based and clinical studies demonstrate significant stage-dependent variability in outcomes and tumour biology, reflecting marked biological heterogeneity [4,5].

In older adults, and particularly in the oldest-old population, tumor behavior and clinical outcomes may differ from those observed in younger patients. Recent data suggest that chronological age alone does not uniformly predict oncological prognosis, and that selected elderly patients may experience relatively indolent disease courses depending on tumor biology, host factors, and overall functional status. This observation reinforces the importance of individualized, rather than age-based, clinical decision-making in gastric cancer management [6].

In oncogeriatrics, particularly for the oldest-old population, international guidelines emphasise the importance of comprehensive geriatric assessment (CGA), shared decision-making (SDM) and value-based care that respects patient autonomy and quality of life. The ethical acceptability of declining active oncological treatment when the decision is informed and autonomous is increasingly recognised in contemporary oncogeriatric practice [7-9].

The present case illustrates this approach in a 94-year-old woman with histologically confirmed gastric SRCC who consciously declined oncological treatment and was managed with supportive care alone, remaining functionally independent and socially active for more than one year after diagnosis.

## Case presentation

Initial consultation (29 November 2024)

A 94-year-old woman of Russian ethnicity presented with a 20-year history of epigastric pain, “hunger cramps,” heartburn, and intermittent discomfort, partially relieved by pantoprazole. Her regular therapy included carvedilol ½ tablet in the morning, lercanidipine 5 mg, candesartan, and enteric-coated acetylsalicylic acid 75 mg daily. Family history revealed cardiovascular diseases and a paternal hematological malignancy (as reported).

On examination, body mass index (BMI) was 26 kg/m², heart rate 103 bpm, and blood pressure 155/90 mmHg; the abdomen was soft with mild epigastric tenderness.

**Figure 1:**
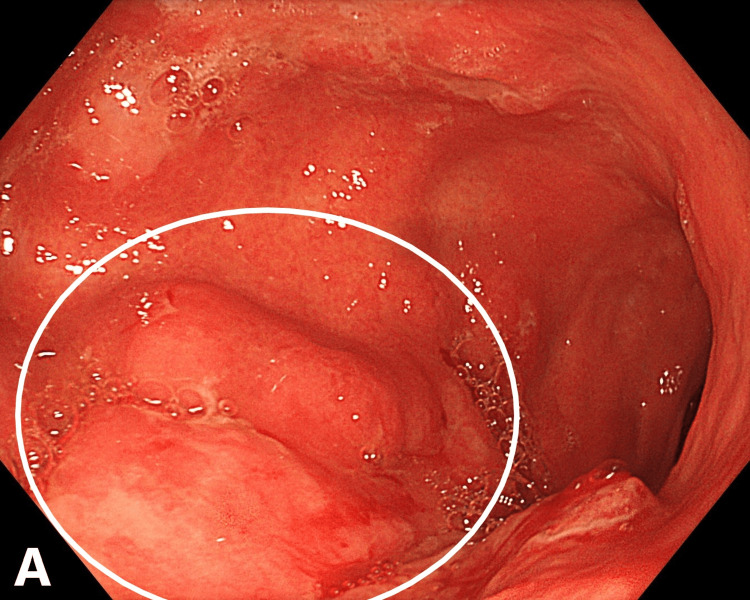
Infiltrative-ulcerative gastric lesion with edema and mucosal hyperemia (endoscopy, November 2024). The circle highlights the area of the infiltrative-ulcerative lesion.

Upper endoscopy performed on November 29, 2024, revealed cardial insufficiency and distal catarrhal esophagitis. The gastric mucosa appeared moderately edematous and hyperemic. An infiltrative-ulcerative lesion with an irregular nodular surface, poorly defined margins, and contact bleeding was visualized in the middle and lower third of the stomach, and multiple biopsies were obtained (Figure 1).

**Figure 2:**
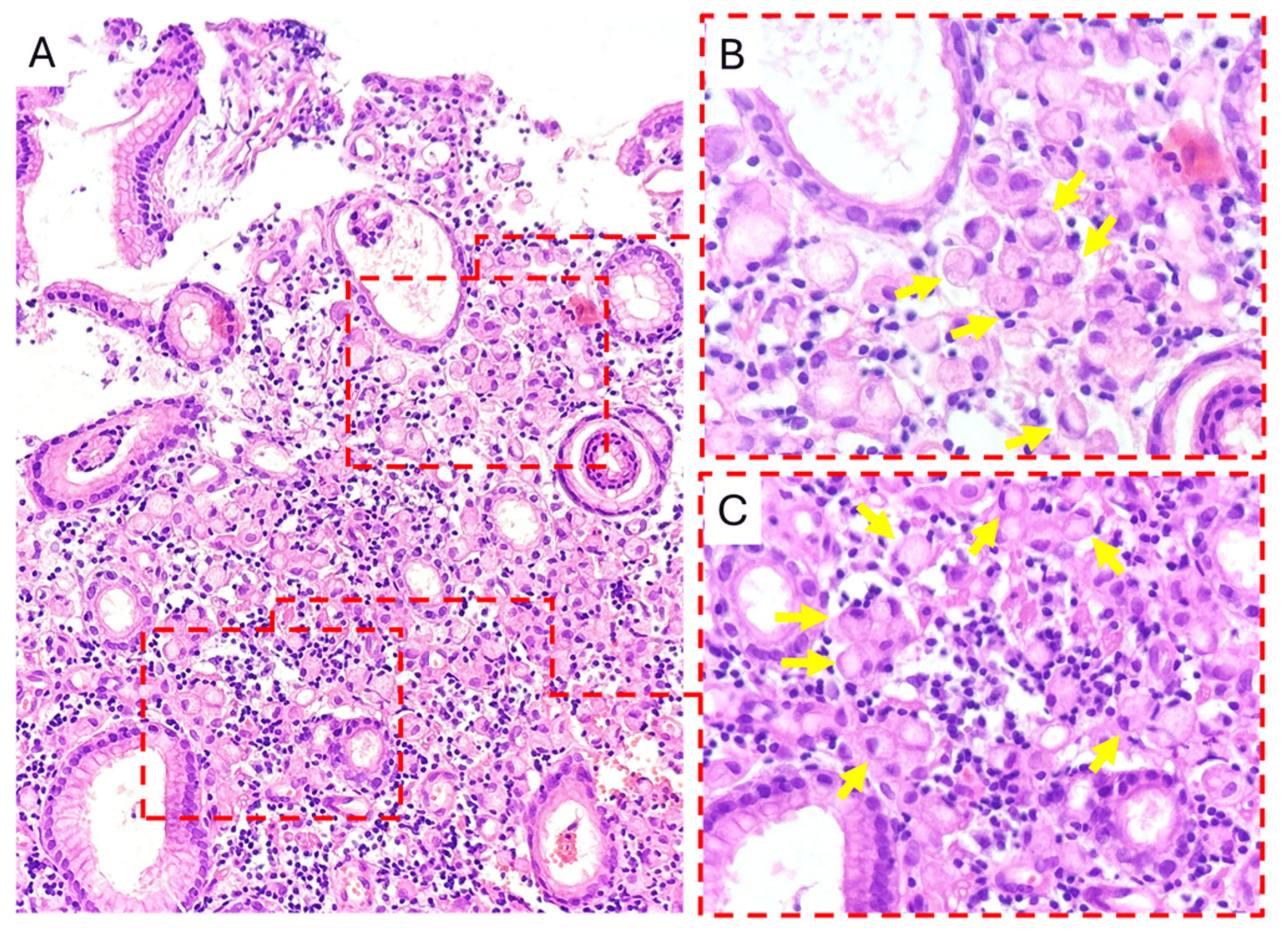
Histopathological features of signet-ring cell carcinoma of the stomach. Hematoxylin and eosin staining. (A) Low-power view (×200) showing diffuse tumoral infiltration by poorly cohesive cells with mucin-rich cytoplasm. Dashed boxes indicate areas magnified in panels B and C. (B, C) High-power views (×400) demonstrating classic signet-ring cells (yellow arrows) characterised by abundant intracytoplasmic mucin displacing the nucleus eccentrically. Fragments of gastric glandular epithelium and surface foveolar epithelium (absence of goblet cells) confirm gastric origin.

Histopathological examination demonstrated diffuse tumoral infiltration of poorly cohesive cells with mucin-rich cytoplasm and eccentrically displaced nuclei, consistent with signet-ring cell carcinoma (ICD-O-3.2 code 8490/3) [1,10]. The histology image corresponding to these findings is provided (Figure 2).

Baseline laboratory investigations, including hemoglobin, iron indices, inflammatory markers, and tumor markers, are summarized in Table 1. 

**Table 1:** Baseline laboratory parameters and reference ranges. Laboratory findings at initial presentation demonstrating mild microcytic anaemia and elevated erythrocyte sedimentation rate. Tumour markers and N-terminal pro-B-type natriuretic peptide (NT-proBNP) were within acceptable limits for the patient’s age and comorbidities. No biochemical evidence of active tumor-related systemic inflammation was present at baseline.

Parameter	Result	Reference range
Hemoglobin	117 g/L	115–155 g/L
Mean corpuscular volume (MCV)	76.5 fL	73–101 fL
Mean corpuscular hemoglobin (MCH)	24.4 pg	26–34 pg
Ferritin	15.86 ng/mL	15–150 ng/mL
White blood cells (WBC)	11.96 ×10⁹/L	4.5–10.2 ×10⁹/L
Platelets	392 ×10⁹/L	142–424 ×10⁹/L
Erythrocyte sedimentation rate (ESR)	56 mm/h	2–30 mm/h
Creatinine	97 µmol/L	45–84 µmol/L
NT-proBNP	305.8 pg/mL	<114.5 pg/mL
Carcinoembryonic antigen (CEA)	1.38 ng/mL	<5.0 ng/mL
CA 19-9	5.29 U/mL	<27 U/mL
CA 72-4	1.35 U/mL	<6.9 U/mL

**Figure 3:**
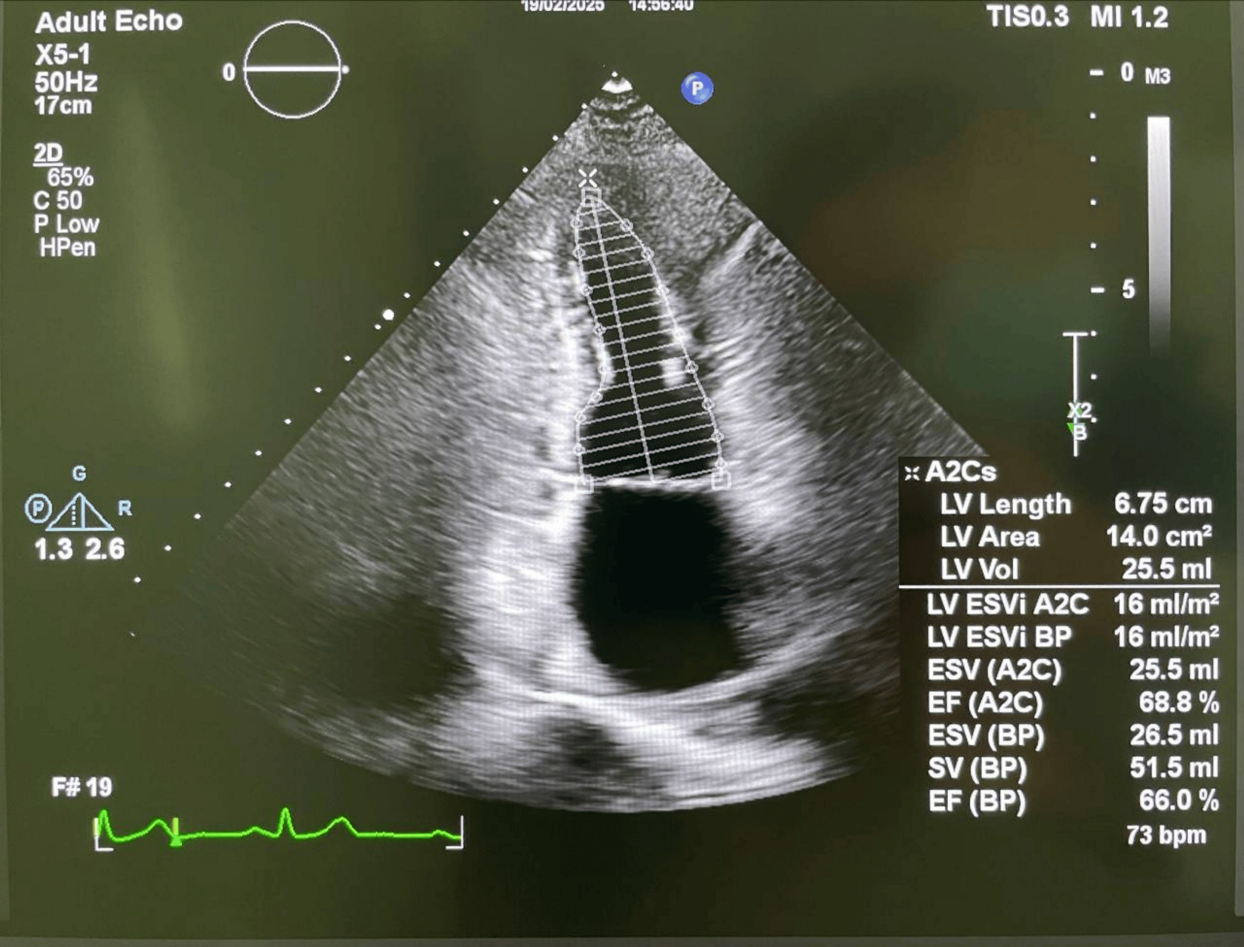
Apical two-chamber echocardiographic view demonstrating left ventricular systolic function. The image shows the endocardial borders traced for biplane Simpson method calculation, with a left ventricular ejection fraction of 66%. Mild concentric remodelling and preserved systolic function are noted.

**Figure 4:**
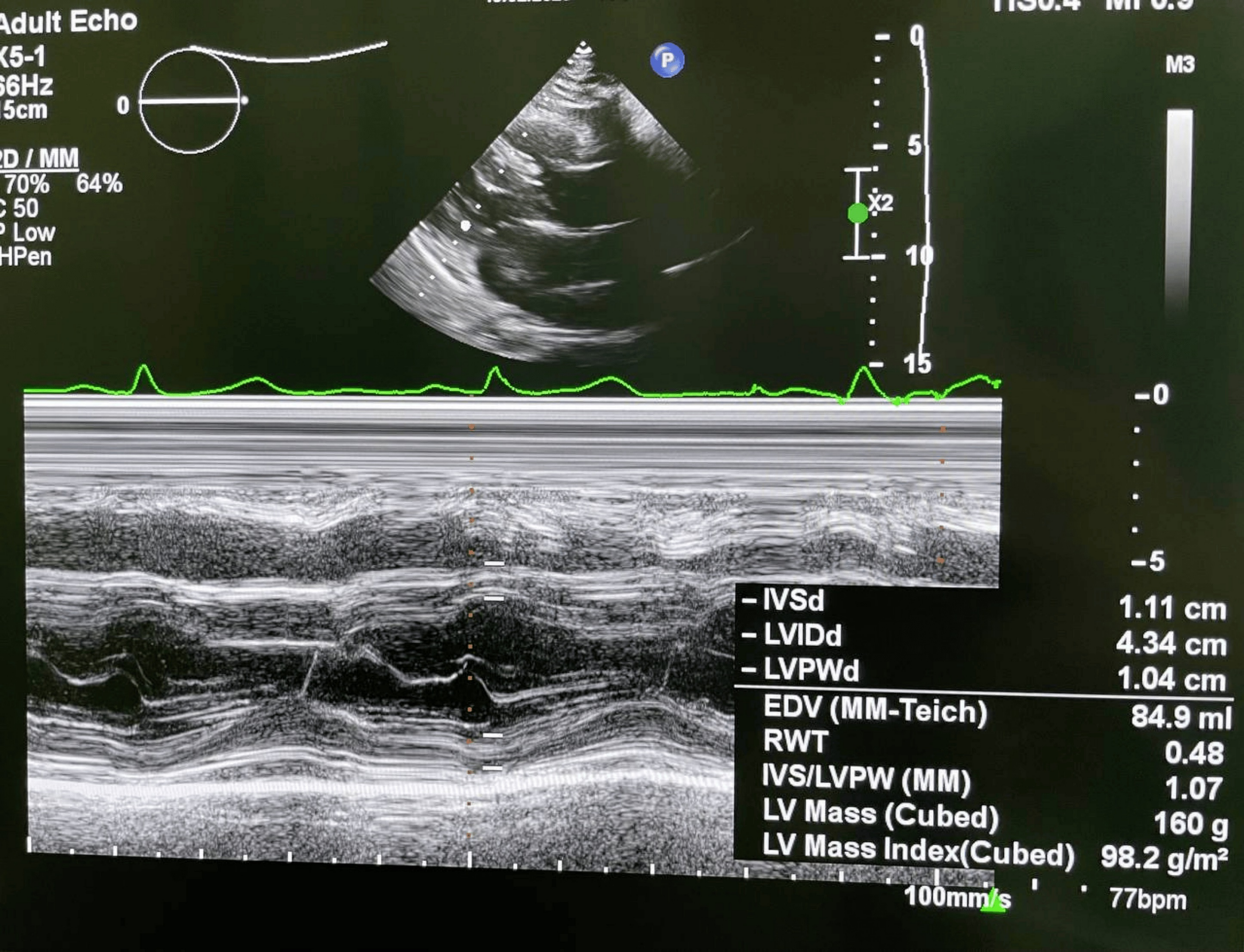
Parasternal long-axis M-mode echocardiogram. Measurements demonstrate normal interventricular septal thickness (IVSd), posterior wall thickness (LVPWd), and end-diastolic diameter (LVIDd), consistent with concentric remodelling. Left ventricular mass index is within the age-adjusted reference limits. No signs of congestive heart failure.

Transthoracic echocardiography showed a left ventricular ejection fraction of 66%, concentric remodeling, mild tricuspid and pulmonary regurgitation, and no signs of congestive heart failure. A representative echocardiographic image is provided (Figures 3, 4). These findings document preserved cardiac function, supporting the patient’s functional resilience and informing oncogeriatric decision-making.

After a detailed discussion of the diagnosis, prognosis, and treatment options, the patient consciously declined surgical or chemotherapeutic intervention and opted for supportive management. Therapy included pantoprazole 40 mg/day (later reduced to 20 mg/day), sucralfati 1 g three times daily for eight weeks, rebamipidi 100 mg three times daily for eight weeks, and a single 500 mg intravenous dose of ferric carboxymaltose. Cardiometabolic optimization included bisoprololi 2.5 mg, sacubitrili/valsartani 50 mg twice daily, dapagliflozini 10 mg, and rosuvastatini 5 mg. A soft, fractionated diet was advised.

Follow-up (9 July 2025)

**Figure 5:**
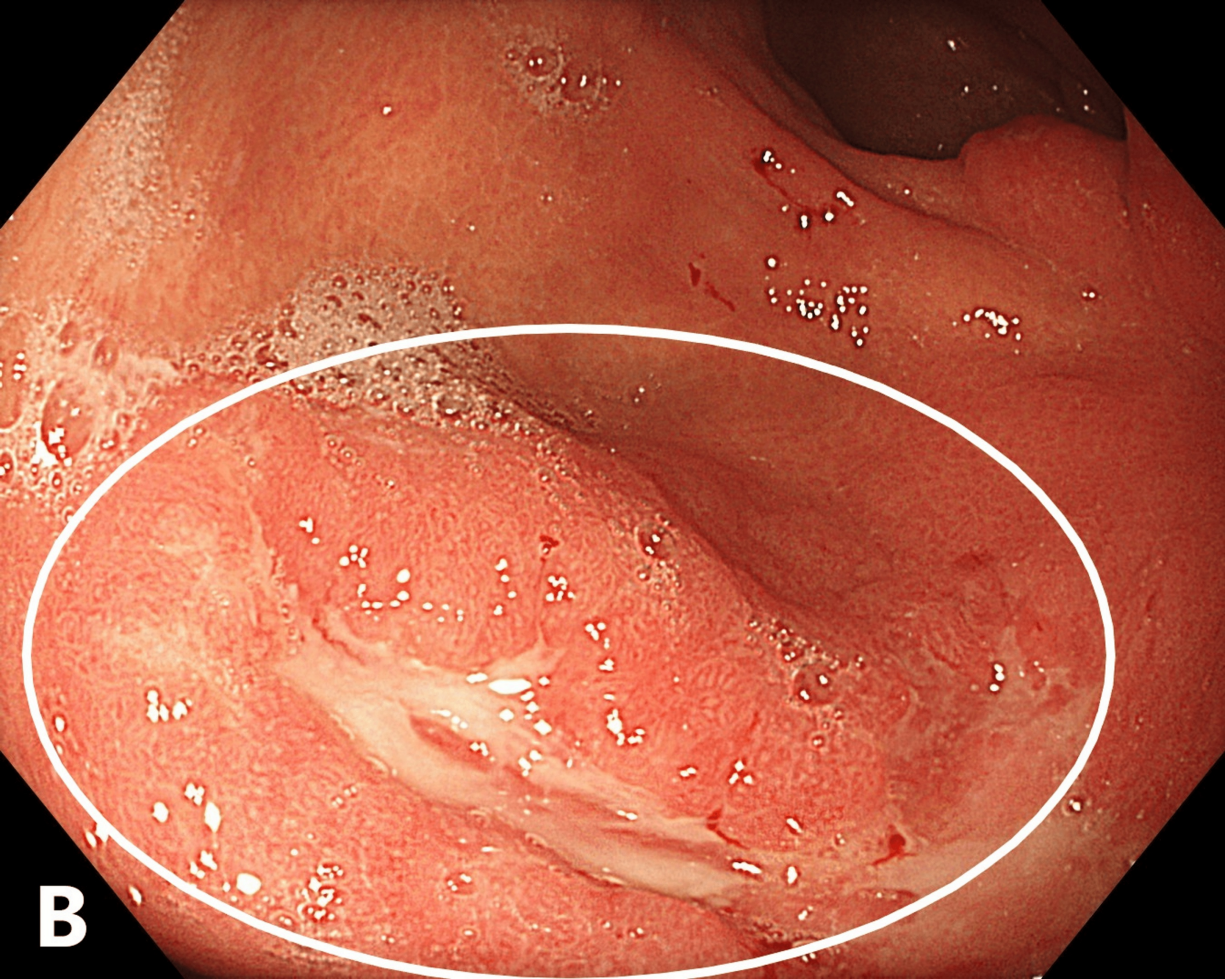
Ulcerated area with fibrinous coating and perifocal edema (endoscopy, July 2025). The circle highlights the persistent ulcerated and scarred region without endoscopic signs of surge-type progression.

Repeat esophagogastroduodenoscopy performed 223 days (approximately 7.4 months) after the initial examination demonstrated persistence of the infiltrative-ulcerative process involving both curvatures, with areas of scarring and moderate gastric deformation, without evidence of surge-type progression (Figure 5).

Follow-up laboratory investigations, including hemoglobin, ferritin, inflammatory markers, and other biochemical parameters, are summarized in Table 2.

**Table 2:** Follow-up laboratory parameters and reference ranges. Laboratory results obtained at follow-up (9 July 2025), showing persistent microcytic anaemia, normal inflammatory biomarkers (hs-CRP and interleukin-6), stable renal function, and lipid profile within acceptable limits for age. HDL-cholesterol remained below the recommended reference threshold for women. HDL: high-density lipoprotein; LDL: low-density lipoprotein, VLDL: very-low-density lipoprotein, hs-CRP: high-sensitivity C-reactive protein

Parameter	Result	Reference range
Hemoglobin	108 g/L	115–155 g/L
Mean corpuscular volume (MCV)	72.6 fL	73–101 fL
Mean corpuscular hemoglobin (MCH)	22.4 pg	26–34 pg
Ferritin	17.52 ng/mL	15–150 ng/mL
White blood cells (WBC)	6.48 ×10⁹/L	4.5–10.2 ×10⁹/L
Platelets	369 ×10⁹/L	142–424 ×10⁹/L
Erythrocyte sedimentation rate (ESR)	35 mm/h	2–30 mm/h
Creatinine	86 µmol/L	44–80 µmol/L
Total cholesterol	3.91 mmol/L	0–5.2 mmol/L
LDL-cholesterol	2.39 mmol/L	0–2.59 mmol/L
HDL-cholesterol	1.25 mmol/L	>1.68 mmol/L*
Triglycerides	1.33 mmol/L	0–1.7 mmol/L
VLDL-cholesterol	0.60 mmol/L	0–1 mmol/L
Uric acid	295 µmol/L	142.8–339.2 µmol/L
hs-CRP	2.02 mg/L	0–5 mg/L
Interleukin-6	3.3 pg/mL	0–7 pg/mL

On examination, BMI was 24 kg/m², heart rate 67 bpm, and blood pressure 120/60 mmHg; the abdomen was soft with mild epigastric tenderness. Weight loss within 6 kg, which may be due to dietary interventions. Functional status assessment demonstrated preserved independence. Handgrip strength measured by dynamometry was 27 kg in the right hand and 23 kg in the left hand. Functional mobility assessed using the Timed Up and Go test was 11 seconds, indicating maintained gait speed and the absence of severe mobility impairment for age.

At the time of manuscript preparation, the patient remains alive, active, regularly socializes with friends, reports good well-being, and occasionally enjoys a small glass of wine in the evening. No signs of clinical decline have been observed (as confirmed by her daughter on November 11, 2025). The documented minimum follow-up since histological confirmation is ≥12 months. This clinical stability may partly reflect age-related differences in tumor-host interactions, as immunobiology in very advanced age differs substantially from that of younger adults.

## Discussion

SRCC, classified by the WHO/IARC as a poorly cohesive carcinoma, is characteristically associated with early peritoneal dissemination and unfavorable outcomes in advanced stages [1-3]. Nevertheless, population-based and clinical evidence highlights substantial heterogeneity in its biological behavior: early-stage SRCC may demonstrate survival rates comparable to non-SRCC adenocarcinomas, while the pace of disease progression varies considerably between patients [4,5]. Mechanistic studies on diffuse gastric cancer further implicate alterations in cell adhesion molecules such as E-cadherin, along with tumor microenvironmental factors and inflammatory signaling pathways, in shaping the spectrum of invasiveness [11].

Importantly, tumor behavior in very advanced age may differ from that observed in younger adults. Age-related changes in immunobiology, including immunosenescence, altered inflammatory signaling, reduced proliferative capacity, impaired telomere maintenance, and changes in tumor-host interactions, may collectively contribute to slower tumor progression in some oldest-old individuals. These mechanisms have been proposed to modulate carcinogenesis and tumor aggressiveness beyond chronological age alone. In the present case, an extremely old woman who consciously declined oncological treatment exhibited an indolent clinical course for at least 12 months, maintaining full functional independence and social activity. In advanced stages, gastric signet-ring cell carcinoma is typically associated with poor outcomes in the general population, with reported five-year survival rates substantially below 30% [11,12]. Against this background, the survival and preserved functional status observed in this 94-year-old patient over more than 12 months highlight the degree of interindividual variability in disease course. Available follow-up laboratory data demonstrated an absence of systemic inflammatory activation, with interleukin-6 and high-sensitivity C-reactive protein remaining within normal reference ranges. The lack of biochemical evidence of active tumor-associated inflammation may partly contribute to the observed indolent clinical course. While isolated reports describe preserved physiological and cognitive resilience among some individuals in the oldest-old category, oncogeriatric decision-making is guided primarily by patient preferences, comorbidities, and a careful evaluation of the risks and benefits of therapy, rather than by assumptions of exceptional biological resistance [7-9].

Importantly, this clinical stability should not be attributed to a single explanatory factor but rather to a multifactorial interaction between tumor biology, host-related aging processes, functional reserve, and care strategy.

This observation illustrates that a supportive, non-aggressive management strategy can be medically rational and ethically sound when the potential survival benefit of intensive therapy is uncertain and the risk of diminished quality of life is substantial. It reinforces the principle that, in geriatric oncology, quality of life may legitimately take precedence over life prolongation when this aligns with patient values and dignity.

Ethical considerations

The decision to forego active oncological treatment was made independently by the patient after full disclosure of her diagnosis, prognosis, and available therapeutic options, including associated risks and potential benefits. This approach is consistent with ESMO/SIOG and American Society of Clinical Oncology (ASCO) recommendations, which emphasize comprehensive geriatric assessment and shared decision-making as essential components of cancer care in older adults [7,8].

Within the framework of value-based and palliative-oriented ethics, the deliberate avoidance of disproportionate medical intervention represents not abandonment of care but an expression of respect for patient autonomy and human dignity [9,13]. This case underscores the ethical imperative to balance clinical action with compassion, ensuring that the goals of care remain aligned with the patient’s informed choices and overall well-being.

## Conclusions

Even histologically aggressive gastric cancers such as signet-ring cell carcinoma may, in select cases, exhibit unexpectedly indolent behaviour in patients of extreme age. In this case, a 94-year-old woman who consciously declined oncological treatment remained alive, active, and socially engaged for more than a year after histological confirmation. Her clinical stability under supportive care highlights the biological heterogeneity of SRCC and the value of geriatric assessment in guiding treatment decisions.

These observations should be interpreted with caution, as conclusions derived from a single case cannot be directly generalized to the broader population of older adults with gastric cancer.

This case illustrates the ethical and clinical legitimacy of prioritising comfort, autonomy, and quality of life over maximal therapeutic intervention when such an approach aligns with the patient’s informed preferences in the oldest-old population. The findings emphasise that in geriatric oncology, the ultimate goal is not the extension of life at any cost, but the preservation of dignity and well-being consistent with the patient’s informed values and choices.
